# A new species of *Pristimantis* (Anura, Craugastoridae) from the Cajas Massif, southern Ecuador

**DOI:** 10.3897/zookeys.751.20541

**Published:** 2018-04-20

**Authors:** Juan C. Sánchez-Nivicela, Elvis Celi-Piedra, Valentina Posse-Sarmiento, Verónica L. Urgiles, Mario Yánez-Muñoz, Diego F. Cisneros-Heredia

**Affiliations:** 1 Laboratorio de Herpetología, Museo de Zoología de la Universidad del Azuay, 24 de mayo 7-77, Cuenca, Azuay, Ecuador; 2 Instituto Nacional de Biodiversidad INABIO, Rumipamba 341 y Av. De los Shyris, Quito, Pichincha, Ecuador; 3 Museo de Zoología, Escuela de Biología, Pontificia Universidad Católica del Ecuador, Quito, Pichincha, Ecuador; 4 Universidad San Francisco de Quito USFQ, Instituto de Zoología Terrestre & Museo de Zoología / Instituto Biósfera / Instituto de Geografía, Quito 170901, Ecuador; 5 King’s College London, Department of Geography, London, UK; 6 University of Central Florida, Department of biology, Florida, USA

**Keywords:** Andes, glandular frog, paramo, *Pristimantis erythros* sp. n., taxonomy, Terrarana, Andes, rana glandular, páramo, *Pristimantis erythros* sp. n., taxonomía, Terrarana

## Abstract

A new species of *Pristimantis* is described from the highland paramos on the eastern slopes of the Cajas Massif, southern Andes of Ecuador, at 3400 m. This new species is characterized by having a distinctive reddish color, cutaneous macroglands in suprascapular region and surfaces of arm and legs, and by lacking dentigerous processes of vomers. The cutaneous macroglands are similar to those exhibited by several species of the *Pristimantis orcesi* group, and may suggest a close phylogenetic relationship. The new species could be a latitudinal substitution of *Pristimantis orcesi* in the southern Andes of Ecuador.

## Abstract (es)

Describimos una nueva especie de *Pristimantis* desde las laderas orientales del macizo El Cajas en los páramos andinos del sur de Ecuador a 3400 m.s.n.m. Esta nueva especie tiene un color rojizo distintivo y se caracteriza por tener macroglándulas cutáneas en varias regiones del cuerpo, de la siguiente manera: la región supraescapular, las superficies del antebrazo, parte superior del brazo, las manos y el borde de las piernas. Además, carece de procesos dentígeros en los vomerinos. Las macroglándulas cutáneas son similares a las presentes en el grupo de *Pristimantis orcesi*, y podrían representar una posición filogenética cercana. La nueva especie puede constituir una sustitución latitudinal de *Pristimantis orcesi* en los Andes sur de Ecuador.

## Introduction

The Andes are one of the major physiographic features on our planet. A heterogeneous mountain system, three geographical separations have been identified in the Andes, based on their different geological, geographical, climatic, and ecosystemic characteristics: northern, central, and southern Andes (Graham 2009). The paramo ecosystem is one of the most distinctive features on the northern Andes, showing remarkable and complex high-altitude flora and fauna communities (Luteyn 1999). Paramos occur on mountain tops above continuous forest line (ca. >3000 m) and below perpetual snow line, mainly in the Andes of Venezuela, Colombia and Ecuador, with outliers on the Andes of northern Peru, and the Central American Cordillera of Costa Rica and Panama. Different vegetation communities are found in paramos, but its general physiognomy is characterized by bush-grasses, rosette and cushion plants, mycrophyllous and dwarf shrubs, and geophytes, with trees usually absent (except for members of the genus *Polylepis*; Luteyn 1999).

Evolution of paramo biodiversity is strongly linked to orogeny and geomorphology, and complex and rich biotas are known to occur across the northern Andes due to their heterogeneous history and topography (Luteyn 1999, Mena and Josse 2000, Sklenář et al. 2011). Paramos show discontinuous distribution, being biogeographic continental islands—isolated one from another by lower areas with different ecologic and physiographic characteristics (Mena and Josse 2000; Mena-Vásconez 2010). Due to their insularity, paramo biota shows important levels of speciation and endemism (Vuilleumier 1970; Fjeldsa 1992; Luteyn 1999; Sklenář et al. 2011; Llambí and Cuesta 2014).

Although amphibian species richness decreases with higher altitude (Navas 2006; Wiens 2007), anurans seem to be more diverse than other ectothermic tetrapods in paramos (Navas 1997, 2006). Due to their low dispersion capacity and high ecophysiological adaptations, anurans are strongly influenced by the insularity of paramos, thus showing high levels of endemism and speciation (Duellman 1979, Lynch 1987). Anuran fauna of the Andes of Ecuador is extraordinarily rich (Duellman 1988), but most collection efforts in the paramo ecosystem have focused on its northern portion. Herein, we describe a distinctive new species of *Pristimantis* from the paramos of the Cajas massif, on the southern section of the Cordillera Occidental, Andes of Ecuador.

## Materials and methods

**Figure 1.**
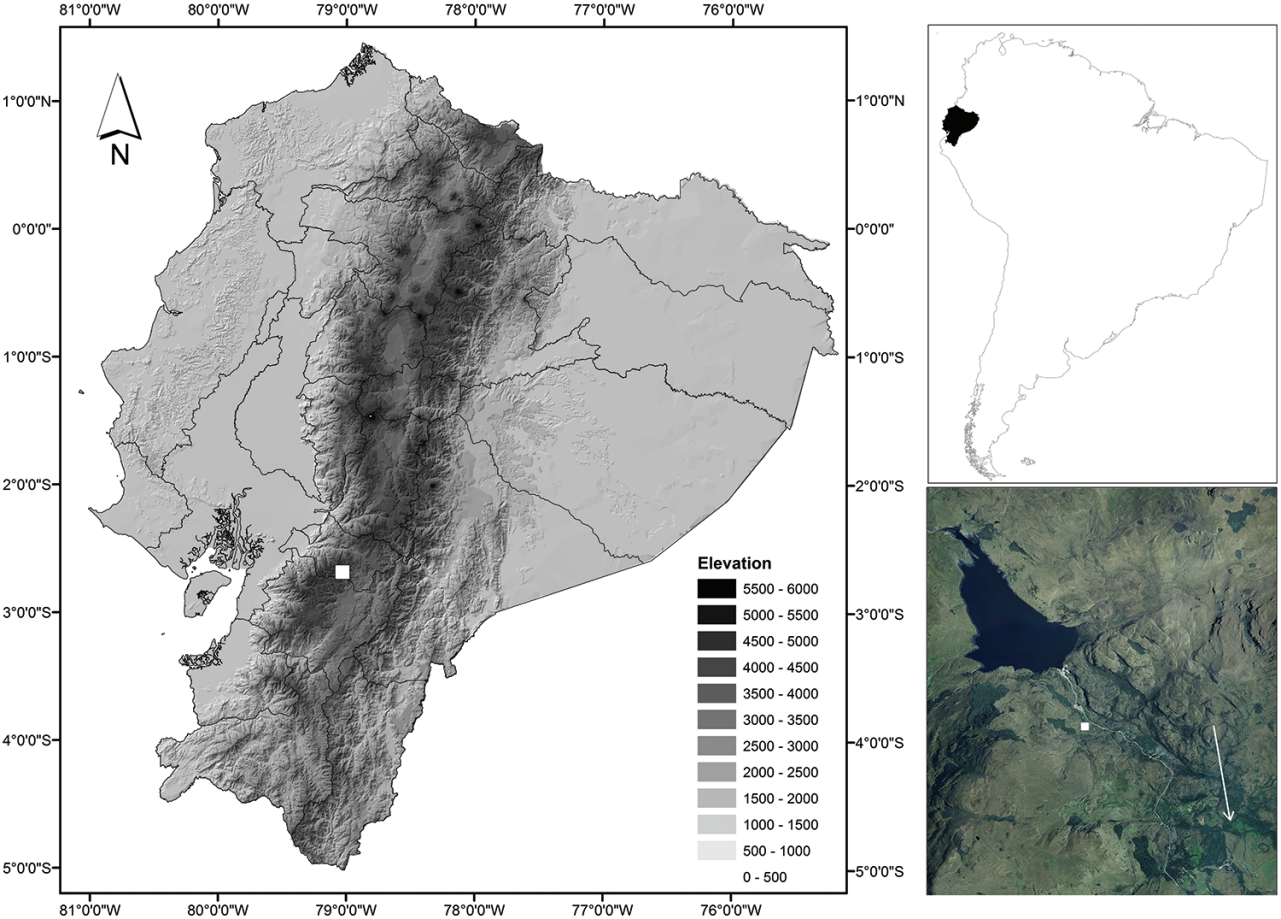
Map of Ecuador showing the type locality of *Pristimantis erythros*, Chanlud (white square), Cajas Massif, province of Azuay, southern Andes of Ecuador. White arrow indicates the direction of Cuenca city, in austral Ecuador.

Collections were made at Chanlud hydroelectric project (Fig. 1), managed by the CELEC hydroelectric company (near ETAPA protected area), northeast of the Macizo del Cajas, province of Azuay, Ecuador. Field work as part of amphibian inventory in the Azuay paramos, and was done across transects methodology (Heyer et al. 1994, Angulo et al. 2006), the sampling were conducted in diurnal (7:00 to 11:00 a.m.) and nocturnal periods (7:00 to 11:00 p.m.). The area has a greater coverage of paramo grassland between 3430 and 3883 meters, with small scattered fragments of forest and shrub. At lower elevation (between 3076 and 3430) the vegetation chances to montane forest, here, have great pressure for deforestation and change land use for agricultural land. Photographs of both living and preserved individuals and their habitat were taken by Juan Carlos Sánchez Nivicela (JCSN). Coordinates and elevations of localities were taken with a GPS data Garmin Etrex 10.

Definitions and terminology follows proposals by Lynch and Duellman (1997) and Duellman and Lehr (2009), except for glandular descriptions that follow Toledo and Jared (1995). Specimens were euthanized with 10% roxicaine, fixed in 10% formalin, and preserved in 75% ethanol. Measurements were taken with digital calipers and rounded to the nearest 0.1 mm, following recommendations by Watters et al (2016). Fingers and toes are numbered preaxially to postaxially from I to IV and I to V, respectively. Comparative lengths of Toes III and V were determined when both were adpressed against to Toe IV; lengths of Fingers I and II were compared when adpressed against each other. Sex was determined by gonadal inspection. Coloration patterns in life, activity patterns, and habitat characteristics were taken from collectors’ field notes and digital photographs. Ecuadorian classification of ecosystems follows the proposal by Ministerio del Ambiente del Ecuador (2013). Examined specimens are deposited at the herpetological collections of Instituto Nacional de Biodiversidad, Quito (**DHMECN**) and Museo de Zoología, Universidad del Azuay, Cuenca (**MZUA**).

## Systematic account

### Pristimantis erythros sp. n.

Taxon classificationAnimaliaAnuraCraugastoridae

http://zoobank.org/DE8E6EBB-37C8-4342-A5F9-5F9A00C2EAC9

#### Common name.

English: Blood Rain Frog. Spanish: Cutín de Sangre

#### Holotype.

**Figure 2.**
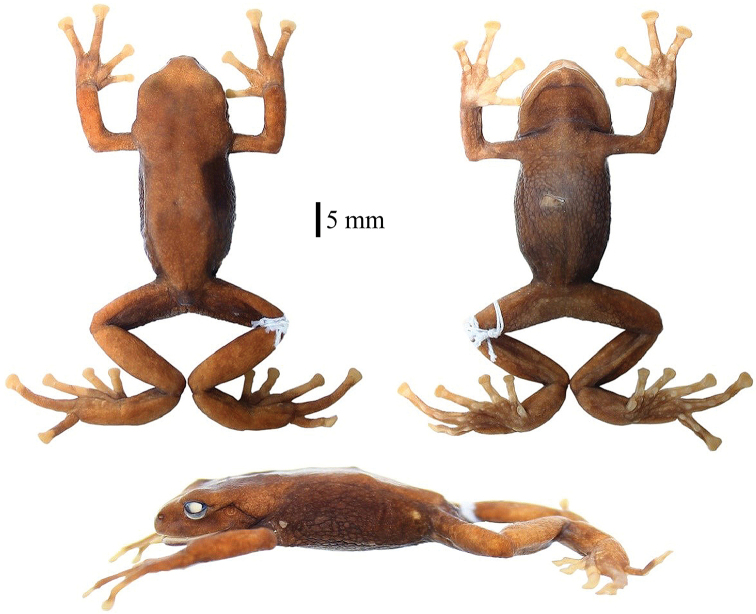
Dorsal, ventral and lateral views of holotype of *Pristimantis erythros* sp. n. (adult female, DHMECN 12103, SVL 39.1 mm) in preservative.

**Figure 3.**
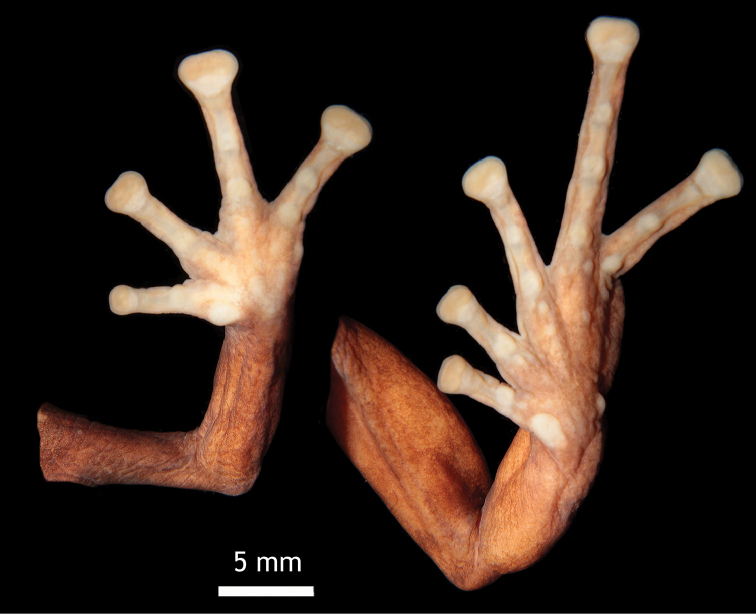
Detail of ventral view of hand and foot of the holotype of *Pristimantis erythros* sp. n. (DHMECN 12103).

**Figure 4.**
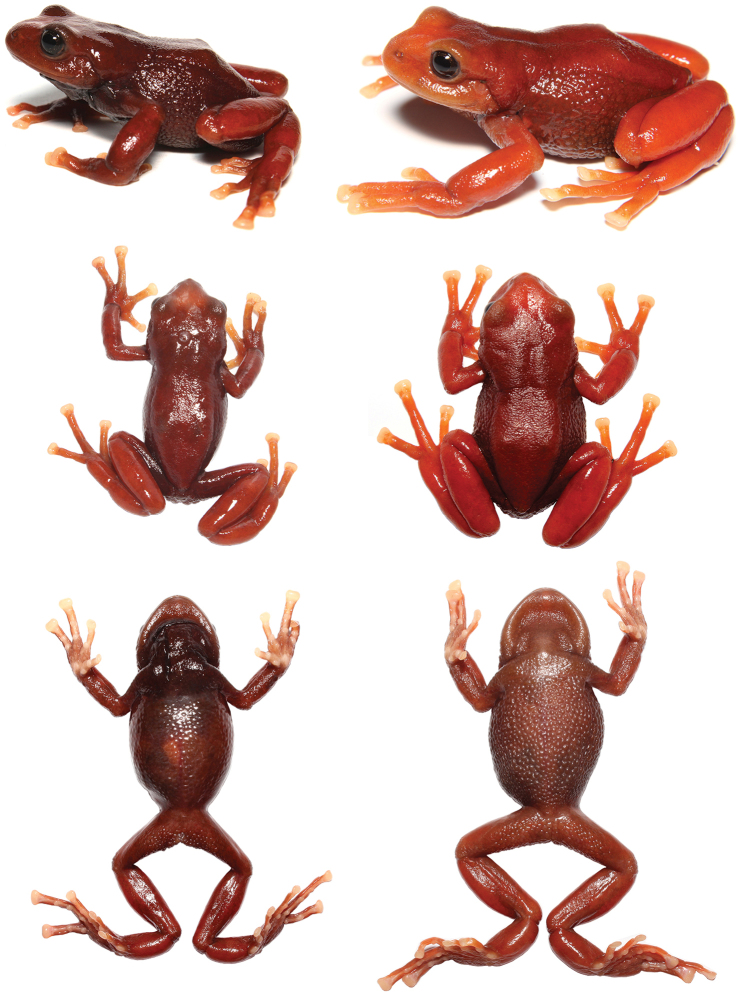
Lateral, dorsal and ventral views of living specimens of *Pristimantis erythros*. Left: Male paratype (DHMECN 12102, SVL: 37.1 mm); right: Female holotype (DHMECN 12103, SVL: 39.1 mm).

DHMECN 12103 (field series JCS.317); (Figs 2–4), an adult female collected at Chanlud, (02°40'57.30"S, 79°1'59.21"W, 3449 m), parroquia Chiquintad, cantón Cuenca, provincial de Azuay, República del Ecuador by JCSN, Verónica Urgilés, Elvis Celi, Valentina Posse and Cristian Nieves, in October 2014.

#### Paratopotypes (11 specimens).

DHMECN 12102, MZUA.AN.1355 adult male; MZUA.AN.1347, MZUA.AN.1348, MZUA.AN.1351, adult females; MZUA.AN.1350, subadult male; MZUA.AN.1349, MZUA.AN.1352, MZUA.AN.1353, subadult females;

MZUA.AN.1342, MZUA.AN.1343 juveniles, collected between October and November 2014 at the type locality.

#### Diagnosis.

*Pristimantis erythros* differs from other species of the genus by the combination of the following characters: (1) Skin on head and dorsum granular, flanks and venter areolate with low warts; dorsolateral folds absent; discoidal fold weakly defined; (2) tympanic membrane and annulus present and visible, rounded, ca. 50% of eye diameter, upper half covered by parotoid macrogland; (3) snout short, rounded in dorsal and lateral views; (4) upper eyelid without tubercles, interorbital distance wider than width of upper eyelid (40%); cranial crests absent; (5) dentigerous process of vomers absent; (6) vocal slits and sacs present in males, nuptial pads absent; (7) Finger I shorter than II; discs laterally expanded with dilated pads and narrow fringes, (8) fingers with coarse lateral cutaneous fringes; (9) low ulnar warts in ventral view; radioulnar macroglands covering the upper surfaces of forearm; (10) heel and tarsus lacking tubercles; paracnemid macroglands on upper surfaces of legs, tarsi, and Toes IV and V; (11) inner metatarsal tubercle oval, not prominent, twice as large as outer metatarsal tubercle, outer metatarsal tubercle rounded and low, supernumerary tubercles low and indistinct; Toe V longer than III, disc of Toe III reaches distal border of penultimate subarticular tubercle on Toe IV, disc on Toe V reaches distal border of distal subarticular tubercle on Toe IV; (12) toes with conspicuous lateral fringes, extend to base of fingers, webbing absent; toe pads as large as or slight larger than those on fingers; (13) in life, dorsum uniformly burgundy, red to orange-red (reddish brown to burgundy in preserved) ; flanks, posterior surfaces of legs, groin, throat and venter crimson (dark reddish brown in preserved); iris dark brown with thin golden reticulations; ventral surfaces of hands and feet pinkish cream; (14) SVL in adult females 38.8–42.6 mm (*x̄* = 40.3, n = 4), in adult males 36.8–37.1 mm (*x̄* = 36.7, n = 2).

#### Comparisons.

**Figure 5.**
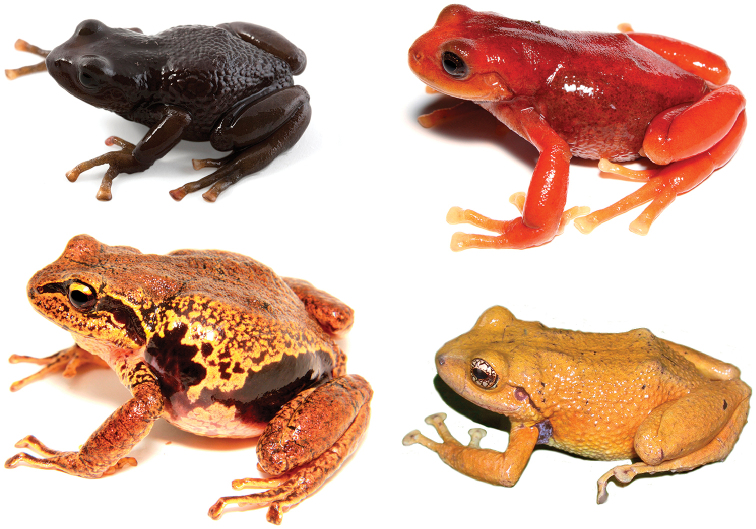
Comparison of *Pristimantis erythros* (top right) with *Pristimantis orcesi* (top left), *Pristimantis pycnodermis* (below left), and *Pristimantis loujosti* (below right).

(Fig. 5) *Pristimantis erythros* differs from all other *Pristimantis* by its conspicuous red coloration in life (reddish brown in preservative), areolate flanks and belly with low warts, cutaneous macroglands: parotoid, paracnemid, and radioulnar; and absence of dentigerous processes of vomers. The distinctive macroglands are also known to be present in *P. orcesi* (Lynch), *P. pycnodermis* (Lynch), and *P. loujosti* Yánez-Muñoz, Cisneros-Heredia & Reyes-Puig. It has a similar external appearance. *Pristimantis orcesi* differs from *P. erythros* by its uniform black to dark brown dorsum in life, areolate skin on dorsum and flanks, low parotoid macrogland and thin paracnemid and radioulnar macroglands on arm and thigh respectively, also *P. orcesi* inhabits paramos on the northern section of Cordillera Occidental and inter-Andean depression of the Andes of Ecuador. *Pristimantis pycnodermis* differs by having low cranial crests, the presence of dentigerous processes of vomer, dark canthal, tympanic marks, and green or brown color with large black spots on the flanks; it inhabits paramos in the southern section of Cordillera Oriental of the Andes of Ecuador. *Pristimantis loujosti* differs by its subacuminate snout in dorsal view, large dentigerous processes of vomers, light orange dorsum, black spots on hidden surfaces of limbs, and light iris with dark reticulation.

#### Description of holotype.

Adult female (Fig. 2), head as wide as the body, slightly wider than long, 8% of SVL; snout short, rounded in dorsal and lateral views, *canthus rostralis* rounded, loreal region concave, nostrils laterally protruding, interorbital area flat, wider than upper eyelid, upper eyelid 15% of interorbital distance; cranial crests absent; parotoid macroglands covering 65% suprascapular dorsal muscle; tympanic membrane differentiated from surrounding skin, evident and rounded ¾ tympanic annulus, laterally directed, upper quarter covered by parotoid macrogland on *cucularis* muscle, tympanum diameter 52% of eye diameter; choanae large and rounded, not covered by palatal floor or maxillary arch; dentigerous processes of vomers absent; tongue broader than long, wider in posterior region, 25% attached to mouth floor.

Skin of dorsum granular without tubercles; dorsolateral folds absent; ventral surface areolate. Discoidal fold weakly defined in ventral view; cloacal region short, and covered by small and pronounced warts (Fig. 2). Ulnar warts slow, radioulnar macrogland covering dorsal surface of arm, forearm and hand; palmar tubercles large, external palmar tubercle, slightly larger than inner, inner palmar tubercle oval; supernumerary tubercles pronounced; subarticular tubercles expanded in dorsal and lateral view; fingers with lateral cutaneous fringes, without interdigital membranes; Finger I shorter than Finger II, discs expanded laterally, all fingers with well-defined circumferential grooves (Fig. 3).

Hind limbs robust, tibia length 44% SVL; heel and external border of tarsus without tubercles, covered dorsally and ventrally by paracnemid macroglands; inner tarsal fold absent; inner metatarsal tubercles oval, twice as larger than the external metatarsal tubercle; supernumerary tubercles present, rounded and flattened; toes with lateral cutaneous fringes; basal membrane absent between toes; foot disks same size as those of hand, laterally expanded from fingers I–IV; relative length of toes 1<2<3<4>5; Toe IV larger than Toe III (Fig. 3).

#### Measurements of holotype (in mm).

Snout-vent length 39.1; head length 10.8; head width 13.8; eye diameter 3.4; eye-nostril distance 3.5; interorbital distance 5.8; internarial distance 3.5; tympanum diameter 1.9; upper eyelid width 2.8; tibia length 17.5; foot length 20.7; hand length 14.5.

#### Coloration of holotype in life.

Dorsum dark red with slightly lighter shades on head and limbs; dark red on venter. Tips of fingers and toes pinkish cream in dorsal view; ventral surfaces of hands and feet, creamy pink. Iris homogeneously dark brown, with thin golden reticulations (Fig. 4).

#### Coloration of the holotype in alcohol.

Dorsum reddish brown, flanks, posterior surfaces of thighs, venter, and throat dark reddish brown. Dorsal surfaces of fingers pinkish cream; ventral surfaces of hands and feet, creamy pink (Fig. 2).

#### Variation.

Morphometric variations of the type series are presented in Table 1. The color variation is the change of tonality that goes from dark red to clear (Fig. 4).

#### Etymology.

The specific epithet *erythros* is derived from the Greek word for red, in allusion to the distinctive coloration of this species.

**Table 1.** Measurements (in mm) of the type series of *Pristimantis erythros* sp. n. All specimens are adults, range is followed by mean ± stander deviation in parentheses. Abbreviations: SVL = snout vent length, HL = head length, HW = head width, ED = eye diameter, EN = eye-nostril distance, IOD = interorbital distance, IND = internarinal distance, UEW = upper eyelid width; TD = tympanum diameter, HAL = hand length, Finger IV disk width = Fin4DW, TL = tibia length, FL = foot length, Toe IV disk width = Toe4DW.

Measurement	Adult Female	Adult Male
	*N* = 4	*N* = 2
SVL	38.8–42.6 (40.2 ± 1.7)	36.7–37.0 (36.9 ± 0.2)
EN	2.7–3.5 (3.2 ± 0.3)	3.1–3.4 (3.3 ± 0.2)
HL	10.6–13.7 (11.5 ± 1.4)	11.5–11.8 (3.3 ± 0.2)
HW	13.2–14.7 (13.7 ± 0.6)	12.6–13.3 (12.9 ± 0.5)
IOD	4.7–5.8 (5.1 ± 0.5)	4.2–5.4 (4.8 ± 0.9)
IND	3.1–3.5 (3.3 ± 0.9)	3.3–3.4 (3.3 ± 0.1)
TL	16.8–17.5 (17.1 ± 0.3)	15.9–16.6 (16.3 ± 0.5)
FL	19.5–21.1 (20.2 ± 0.8)	18.4–18.5 (18.4 ± 0.1)
HAL	13.0–14.4 (13.8 ± 0.6)	12.3–12.8 (12.5 ± 0.4)
TD	1.7–1.9 (1.8 ± 0.1)	1.7–1.8 (1.8 ± 0.1)
ED	3.4–4.2 (3.7 ± 0.4)	2.9–3.8 (3.3 ± 0.7)
UEW	2.6–3.5 (3.0 ± 0.4)	3.1–3.3 (3.2 ± 0.1)
Fin4DW	2.3–3.5 (2.6 ± 0.3)	2.2–2.4 (2.3 ± 0.2)
Toe4DW	2.2–2.7 (2.5 ± 0.2)	2.1–2.3 (2.2 ± 0.1)

#### Distribution, natural history, and extinction risk.

**Figure 6.**
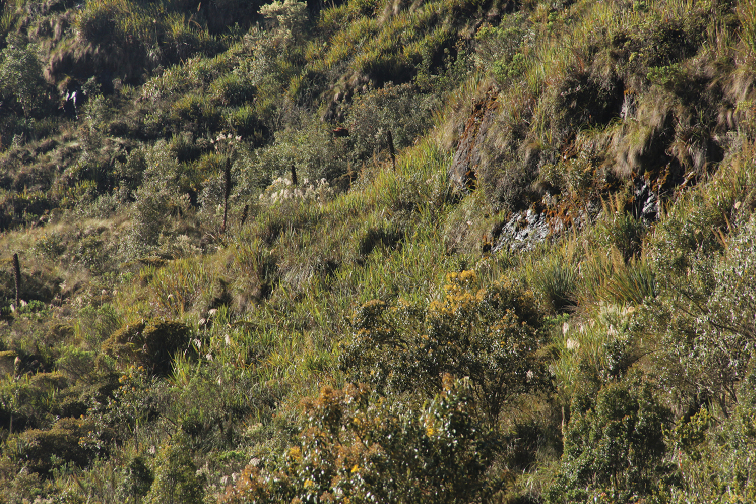
Habitat of *Pristimantis erythros* in type locality.

*Pristimantis erythros* is only known from its type locality in the Cajas Massif. The area is covered by paramos dominated by grassland and shrubs, between 3450 and 3500 m (Fig. 6). Specimens were collected mainly in terrestrial bromeliads (*Puya hamata*) and grasses (*Neurolepis villosa*), near to small streams. Vocalizations were heard (but unrecorded) during daytime hours from 08h00 to 11h00 and from 17h00 to 19h00. Active individuals were observed from dusk until approximately 21h00, afterwards activity decreased. The new species was recorded in sympatry with Pristimantis aff. cryophilius, P. aff. orestes and P. aff. riveti.

The Paramos on the Cajas Massif (221000 h. approx.) appear well preserved. Part of its extension includes the Cajas National Park (28544 h). However, the continued changes on land cover and land use occurring in several areas over the massif on the buffer area of the national park and not protected nearest regions are leading to habitat loss (Hofstede et al. 2002). During a period of four (4) years (2014–2017), twenty six (26) localities in suitable regions (2500–3500 m) on the Cajas Massif were surveyed, no additional records of this new *Pristimantis* were added during these excursions mentioned above. It is probable that *P. erythros* inhabit only a single locality in an area of less than 1 km^2^. Finally, based on the small area of occupancy that might be restricted to the type locality which it is not under conservation in a protected area, we suggest that, it should be classified as Critically Endangered (CR) under the UICN criteria B1,B2ab(i,ii,iii,iv) (IUCN 2001).

## Discussion

At least 50 species of anurans, including *Pristimantis erythros*, are currently known to inhabit the paramos of Ecuador (Table 2). The distribution of these species is fairly even along Andes, with 34 species recorded on the paramos of Cordillera Occidental (21 spp. on the northern part, 17 spp. southern part), and 36 species on the paramos of Cordillera Oriental (19 spp. on the northern section, 21 spp. southern section). Our data show that terrestrial frogs of the genus *Pristimantis* make a significant proportion of the amphibian fauna in Ecuadorian paramos (50–58% on each mountain range; slightly higher than calculations by Navarrete et al. 2016). The lowest species richness of *Pristimantis* occurs in the southern paramos (7 spp. on Cordillera Occidental and 10 spp. on Cordillera Oriental), probably referred as collection bias since several species from this section remain undescribed.

**Table 2.** Amphibians of the paramos from the Andes of Ecuador (above 3000 m). Abbreviations: N = northern section, S = southern section (sections are approximately divided by 1.5°S latitude). Nominal species that may be complexes (including more than one cryptic species) are marked with an asterisk.

Species	Cordillera Occidental		Cordillera Oriental		Source
	N	S	N	S	
*Atelopus bomolochos*					Peters (1973)
*A. exiguus*					Coloma et al. (2000)
*A. ignescens*					Coloma et al. (2000)
*A. nanay*					Coloma (2002)
*A. pastuso*					Coloma et al. (2010)
*A. petersi*					Coloma et al. (2007)
*A. podocarpus*					Coloma et al. (2010)
*Osornophryne angel*					Yánez-Muñoz et al. (2010)
*O. antisana*					Hoogmoed (1987)
*O. talipes*					Cannatella (1986)
*Centrolene buckleyi* *					Lynch and Duellman 1973
*Hypodactylus brunneus*					Lynch (1975)
*H. peraccai*					Lynch (1975)
*Lynchius flavomaculatus*					Lynch (1975)
*Pristimantis buckleyi* *					Lynch (1981)
*P. cajamarcensis*					Lynch and Duellman (1997)
*P. cryophilius* *					Lynch (1979)
*P. cryptomelas*					Lynch (1979)
*P. curtipes* *					Lynch (1981)
*P. devillei* *					Lynch and Duellman (1980)
*P. erythros*					This work
*P. festae*					Lynch and Duellman (1980)
*P. gentryi*					Lynch and Duellman (1997)
*P. gualacenio*					Urgilés et al. (2014)
*P. huicundo*					Guayasamin et al. (2004)
*P. leoni* *					Lynch and Duellman (1997)
*P. lymani*					Lynch and Duellman (1997)
*P. mazar*					Guayasamin and Arteaga (2013)
*P. modipeplus*					Lynch (1981)
*P. myersi*					Lynch (1981)
*P. ocreatus*					Lynch (1981)
*P. orcesi* *					Lynch (1981)
*P. orestes* *					Lynch and Duellman (1997)
*P. ortizi*					Guayasamin et al. (2004)
*P. philipi*					Lynch and Duellman (1995)
*P. phoxocephalus* *					Lynch and Duellman (1997)
*P. pichincha*					Reyes-Puig and Páez-Rosales (2016)
*P. pycnodermis*					Lynch and Duellman (1980)
*P. riveti* *					Lynch (1979)
*P. thymelensis*					Lynch (1981)
*P. unistrigatus* *					Lynch (1981)
*Hyloxalus anthracinus*					Coloma (1995)
*H. jacobuspetersi*					Coloma (1995)
*H. vertebralis*					Coloma (1995)
*Gastrotheca espeletia*					Duellman (2015)
*G. litonedis* *					Duellman (2015)
*G. pseustes* *					Duellman (2015)
*Hyloscirtus larinopygion*					Duellman and Hillis (1990)
*Telmatobius niger*					Trueb (1979)
*T. vellardi*					Trueb (1979)

The Cajas Massif has one of the most particular landscapes in the Ecuadorian Andes. The massif was glaciated during the Pleistocene (Hastenrath 1981, Clapperton 1993), and its current physiography includes more than two hundred glacial lakes, interconnected ridges and peaks, and numerous broad hanging valleys (Coblentz and Keating 2008). The Cajas Massif holds the largest continuous paramos on the Cordillera Occidental of Ecuador. These paramos are separated from all surrounding highlands by the River Cañar basin (north), the River Jubones basin (south), and the intra-Andean basin of Paute (east). At least four species of anurans are endemic to the paramos of the Cajas Massif: *Atelopus exiguus*, *A. nanay*, *Pristimantis erythros*, and *P. philipi*. In fact, the Cajas Massif seems to be an important endemic area for biodiversity (Barnett 1997), with several endemic species of plants (incl. at last nine species of the genus *Valeriana*, Sklenář and Jørgensen 1999), birds (incl. *Metallura baroni* and *Xenodacnis* sp., Astudillo et al. 2015, and mammals (incl. *Chibchanomys orcesi*, Jenkins and Barnett 1997).

*Pristimantis erythros* share conspicuous cutaneous macroglands on its body and extremities with *P. orcesi*, *P. pycnodermis*, and *P. loujosti*. *Pristimantis erythros* is most similar to *P. orcesi*, from which it differs by its coloration and morphology, and has a significant biogeographic separation due to the isolation of the Cajas Massif from other paramos. Phylogenetic relationships of *P. erythros* are still uncertain, and due to the lack of additional evidence (e.g., molecular data), we refrain to assign *P. erythros* to any species group. Although *P. erythros* and *P. orcesi* may be related, the *Pristimantis orcesi* species-group is not a monophyletic group (Hedges et al. 2008, Padial et al. 2014). We do not discard the possibility that *P. erythros* replaces latitudinally *P. orcesi*.

## Supplementary Material

XML Treatment for Pristimantis erythros
